# Evolutionary models of lateralization: Steps toward stigmergy?

**DOI:** 10.3389/fnbeh.2023.1121335

**Published:** 2023-02-02

**Authors:** Lucio Tonello, Giorgio Vallortigara

**Affiliations:** ^1^Center for Nonlinear Science, University of North Texas, Denton, TX, United States; ^2^GIOYA HEI, E305, The Hub Workspace, San Gwann, Malta; ^3^Center for Mind/Brain Science, University of Trento, Rovereto, Italy

**Keywords:** lateralization, brain asymmetry, stigmergy, swarm intelligence, evolutionary models, self-organizing systems, complexity

## 1. Introduction

Several theoretical models have been proposed to account for brain and behavioral asymmetry (e.g., Ghirlanda and Vallortigara, 2004; Vallortigara, 2006; Abrams and Panaggio, 2012; Rogers and Vallortigara, 2015; reviews in Ocklenburg and Güntürkün, 2018; Vallortigara and Rogers, 2020).

An interesting approach is based on competition and cooperation in phenotypically asymmetric individuals. Ghirlanda et al. (2009) suggested that population-level lateralization can arise as an evolutionarily stable strategy and that left- and right- lateralized individuals in unequal numbers can be evolutionary stable based solely on the balance between competitive and cooperative interactions. Cooperative activities would favor individuals with the same lateralization (by coordinating physical activities, efficiently using the same tools, etc.). Competitive activities would favor individuals different from the majority who would be able to surprise opponents, adopting behaviors to which opponents are less accustomed (e.g., human left-handers that may hold an advantage in fighting or, in more recent times, in certain sporting activities, review in Frasnelli and Vallortigara, 2018).

More specifically, the model by Ghirlanda et al. (2009) introduces a fitness function, *f(x)* = *a(x)* + *cs(x)*, where *a(x)* accounts for antagonistic interaction (say a competition function) and *s(x)* for synergistic interaction (say a cooperation function); *c* is the control parameter tuning the relative strength between *a(x)* and *s(x)*. The variable *x* is given by the ratio between L and R. The model shows, in an almost fully analytical way, that under proper conditions, there exists an unequal number of equilibrium of left-and right-lateralized individuals.

## 2. A different perspective

The model can be described from a different perspective, by a more numerical approach, which is both probabilistic and evolutionary. It can be viewed as a sort of very simple “*in silico*” simulation of individual lives. The algorithm is reported below.

- Let us take a population (e.g., 10,000 individuals) where each individual can assume one of two possible states, say “L” or “R.” As initial conditions, we can assign state R or L in a totally random way, in any random ratio.
- For each generation (i.e., iteration), each individual has the probability of having a progeny proportional to his/her fitness level (we employ the same fitness function suggested in Ghirlanda et al., 2009). The progeny (if any) will have the same state (L or R) as his parent.
- At each generation, the population would expect to increase because it consists of parents with their possible progeny, but some individuals die in a proportion such that the total number of individuals remains the same (in our example, 10,000 individuals). The distribution of deaths is assumed to be uniform across the population (e.g., if the number of R-individuals is 3 times greater than L-, then when an individual would die, the probability that they are an R- is three times greater than L-).

The algorithm, implemented using R Core Team (2021), leads to the same outcome as in Ghirlanda et al. (2009) who chose the parameter values (*Ka* = *5, Ks* = *1*), so that a(x) decreases more rapidly than s(x) increases, so in the fitness function competition varies more quickly than cooperation. Using these parameters in our model, we observe that after some iterations, the system moves to an equilibrium in the same way, as follows: for *c* <0.68, we have 50% L and 50% R, meaning that function *a(x)* dominates (competition); for 0.68 <*c* <1.57, we have an “unequal number” equilibrium between 50 and 100%, so both *a(x)* and *s(x)* are relevant (a mixture of competition and cooperation); for *c* > 1.57, we have 100% L (or 100% R) meaning that Function*s (x)* dominates (cooperation).

**Figure 1:**
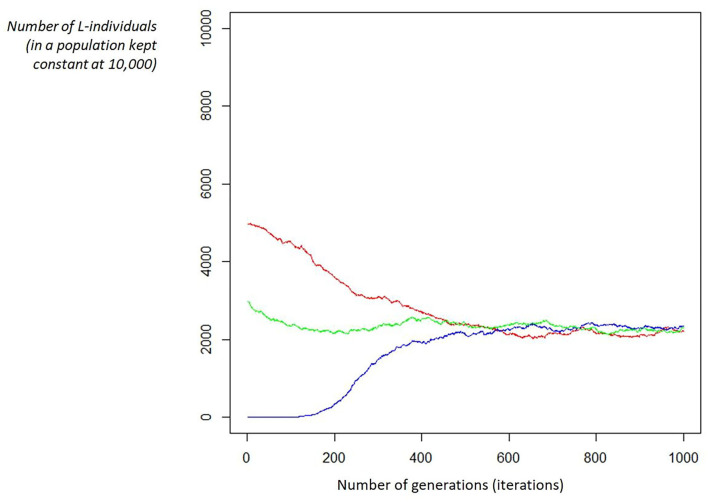
Examples of convergence. Given the fitness function parameters (e.g., c = 0.9, Ka = 5, Ks = 1) and a fixed population (e.g., 10,000 individuals), the ratio between L/R individuals will converge to the same proportion (ruled by the “c” parameter): in this case, c = 0.9 leads to a proportion of 22% of L-individual, whatever the starting ratio (in the figure, 3 starting ratios are shown: red is L = 50.00%, blue is L = 0.05%, and green is L = 30.00%).

The simulation confirms the behavior of the previous model in a detailed way and, in particular, it shows that whatever the initial proportion between L and R, it will converge to the predefined equilibrium imposed by the “c” parameter. We could describe it as a game: putting a group of individuals (large enough) on a desert island, whatever the L/R initial ratio, it will always evolve to the ratio defined by “*c*,” the relative strength between competition and cooperation (refer to Figure 1).

## 3. Discussion

The simulation does not provide in itself any novel outcome, but it describes the dynamics from a different point of view, suggesting new possible interpretations and developments. In particular, it can suggest considering the issue of a single individual perspective.

Once all of the parameters are fixed (e.g., *c* = 0.9, *Ka* = 5, *Ks* = 1), an individual fitness can be evaluated according to the L/R ratio of the full population, i.e., a single individual fitness function *f(x)* has only one input variable *x*, which is the proportion of L and R on the whole population. Now, in a real-life condition, how can the single individual asymmetry be related to the total ratio of L and R, in a numerical sense? Of course, the individual does not have a direct measure of how many left- or right-lateraled individuals are there in the population, but they should obey the fitness rule anyway, and therefore, they should have some form of the estimate of the L and R ratio. How can an individual undergo such an indirect estimate?

Well, we can think of an individual who, in their life, has to deal with a subset of the full population. This subset would be made of the actual individuals they can cooperate or compete with, a kind of neighborhood, which is their actual behavioral environment. It is likely that such an environment, apart from small oscillations, will have quite a similar R-L proportion value as the whole population. Thus, the individual would interact with a portion of the L- and R- population only, which can determine their fitness in line with the other portions of the population. It is in the interaction with others (L or R) with whom an individual exhibits cooperation and competition, and according to the proportion of them they have to deal with, that their fitness would achieve a proper value.

However, this view is only partially convincing. Let us suppose an individual is right-handed (or for other non-human species that it exhibits some lateral bias in the use of eyes, ears, tails whatever, e.g., Vallortigara and Versace, 2017): interacting with many right-handed individuals would lead to cooperating more, according to the number of right-handed individuals they would interact with. Some issues seem still open, e.g., will an individual meet a large enough number of others, so that the L/R proportion will be in line with the whole population?

Now, let us suppose that it is not the direct interaction with others as single individuals but the outcome of being in a biased existing world as a group that would matter. For example, suppose that R is the majority: this would have the effect of having more available, say, tools for manipulation for right-handed individuals. We can imagine this as an environmental change made by R individuals, which is made to be more suitable for R individuals.

In such a view, we can think that the described sport superiority (confrontation) of the left-handed in some disciplines (review in Rogers et al., 2013) can not be (or not only) because of a direct effect on individuals' confrontations but as the outcome of an environmental change: if, say, a famous athlete wins again and again, they will attract many sponsorships and this would impact their fitness. The number of wins is not important, nor the number of one-to-one confrontations, but rather the environmental effect.

We could conceive that the information related to minority and majority, to L/R ratio, could come from actions on the environment by asymmetrical individuals which, in turn, would affect the R and L single individual fitness. In this view, the individual fitness function could be not linked to the “x” ratio because of the frequency of L and R direct interactions or direct mechanisms (such as quorum sensing) but because of experiencing environmental changes affecting the ratio between L and R.

We speculate that this kind of dynamic could be an instance of stigmergy, a term introduced by Grassé to describe a form of indirect communication mediated by modification of the environment that he observed in some species of termites (Theraulaz and Bonabeau, 1999) and since widely suggested to be a pillar of optimization tasks performed by ant colonies and other social insects (Dorigo et al., 2000; Theraulaz, 2014). In this perspective, the L and R equilibrium could express a kind of intelligence (in a mathematical meaning of the term) known as “swarm intelligence.” The view here is that a single L or R individual is just passively and unknowingly undergoing the mechanics of fitness, and there is not a leader in the game imposing an R and L ratio, or a fixed ratio genetically predetermined.

What seems to emerge instead is that the system self-organizes in an intelligent way because it moves autonomously toward an optimal equilibrium—so it is intelligent only when operating as a group.

It could be conjectured as a self-organizing complex system. The individual (in the L/R evaluation) does not know what the equilibrium is. The population as a whole would lead them to a better equilibrium if each individual could communicate with any other in an indirect way through the environmental change in a social way, as a form of stigmergy. It could perhaps be conjectured that whether stigmergy is communicated in a social way, behavioral population, asymmetries are more likely to appear in social species.

## Author contributions

All authors listed have made a substantial, direct, and intellectual contribution to the work and approved it for publication.
